# Effect of Enhanced Hydrolytic Acidification Process on the Treatment of Azo Dye Wastewater

**DOI:** 10.3390/molecules28093930

**Published:** 2023-05-06

**Authors:** Xuehui Xie, Yiting Qin, Shanshan Yang, Yao Sun, Haonan Mo, Hangmi Zheng, Na Liu, Qingyun Zhang

**Affiliations:** 1 Key Laboratory of Textile Science & Technology (Donghua University), Key Laboratory of Pollution Control and Emission Reduction Technology for Textile Industry, College of Environmental Science and Engineering, Ministry of Education, State Environmental Protection Engineering Center for Pollution Treatment and Control in Textile Industry, Donghua University, Shanghai 201620, China; 2 Shanghai Institute of Pollution Control and Ecological Security, Shanghai 200092, China; 3 School of Environment and Surveying Engineering, Suzhou University, Suzhou 234000, China; 4 School of Chemical and Environmental Engineering, Anhui Polytechnic University, Wuhu 241000, China

**Keywords:** biological intensification technology, hydrolytic acidification, co-matrix, azo dye wastewater

## Abstract

The hydrolysis acidification process is an economical and effective method, but its efficiency is still low in treating azo dye wastewater. It is therefore crucial to find more suitable and efficient means or techniques to further strengthen the process of treating azo dye wastewater by a hydrolytic acidification process. In this study, a hydrolytic acidification aerobic reactor was used to simulate the azo dye wastewater process. The change of wastewater quality during the reaction process was monitored, and the deep enhancement effect of single or composite biological intensification technology on the treatment of azo dye wastewater by the hydrolytic acidification process was also explored. Co-substrate strengthening and the addition of fructose co-substrate can significantly improve the efficiency of hydrolytic acidification. Compared with the experimental group without the addition of fructose, the decolorization ratio of wastewater was higher (93%) after adding fructose co-substrate. The immobilization technology was strengthened, and the immobilized functional bacteria DDMZ1 pellet was used to treat the simulated azo dye wastewater. The results showed that the composite technology experimental group with the additional fructose co-matrix had a better decolorization efficiency than the single immobilized bio-enhancement technology, with the highest decolorization ratio of 97%. As a composite biological intensification method, the fructose co-matrix composite with immobilized functional bacteria DDMZ1 technology can be applied to the treatment of azo dye wastewater.

## 1. Introduction

The use of dyes has a long history and is a valuable and important material in our daily life [1]. Azo dyes, as some of the most commonly used industrial dyes, account for more than 60% of the total amount of dyes, and their importance may be further enhanced in the future [2]. Azo dyes are widely used in textile, paper making, and food industries [3,4]. During the treatment of water containing organic pollutants, a large number of by-products can be formed [5]. Under special conditions, azo dyes can also decompose more than 20 kinds of carcinogenic aromatic amine compounds, which can cause changes in the human DNA structure and lead to cancer [6,7,8]. Relevant environmental researchers have noticed for a long time that the molecular structure of azo dyes is very stubborn. In addition, the toxicity of azo dyes and their decomposition products to microorganisms makes the treatment of azo dye wastewater very difficult [9]. At present, various physicochemical or biological methods have been used to treat azo dye wastewater. Among them, the hydrolysis acidification process, as an economical and efficient treatment method, is widely used in the treatment of chemical wastewater, printing, dyeing wastewater, and other refractory wastewater due to its unique advantages [10]. Hydrolysis refers to the biochemical reaction of organic matter outside the cell before it enters the microbial cell. Acidification is a typical fermentation process. The hydrolysis acidification process is developed on the basis of an anaerobic wastewater biological treatment process, which only allows the reaction to stay in the two stages of hydrolysis and acidification and does not enter the subsequent stages of hydrogen production, acetic acid production, and methane production. The hydrolysis acidification process is an economical and effective method. Even if the concentration of dye in wastewater is very low, the presence of dye chromaticity also significantly damages the ecological environment [11]. The acidification process can effectively improve the biodegradability of wastewater while achieving a certain removal rate for chromaticity [12,13], although the general efficiency of this process is still low. It is important to find appropriate means or technologies to further strengthen the process of the hydrolysis acidification process in treating azo dye wastewater. The intensification means mainly include bacterial agent intensification, co-matrix intensification, and immobilization technology intensification [14,15,16]. Bacterial intensification is simple and effective, but it faces the problem of the easy loss of bacteria with water flow. In addition, the addition of bacterial agents and flora will increase the cost of wastewater treatment. Co-matrix enhancement is a method to add simple energy substances, such as small molecule organics, degradation products, or intermediates of target pollutants, into the hydrolysis acidification reaction system, so that microorganisms in it can degrade substances that could not be degraded before [17,18]. Many organic compounds that are difficult to degrade can be degraded through the co-metabolism pathway using small molecule organic compounds as carbon sources. Immobilization technology enhancement refers to a biological technology that retains or restricts free microbial cells in a specific space area through physical or chemical methods and keeps them active for a long time. Immobilized functional bacteria have the advantages of high activity, high stability, low sludge output, etc. [19,20]. This study focuses on strengthening methods such as functional fructose co-matrix strengthening and immobilization of functional bacteria DDMZ1. In addition to studying the strengthening effect of a single method, it also explores the deep strengthening effect of different methods combined to treat simulated azo dye wastewater by the hydrolysis acidification process.

In the early stage, our research studied the acquisition mode, community structure, and characteristics of the functional bacteria group DDMZ1 and found that fructose is the best co-metabolism substrate in the degradation process of reactive black 5 dye. In addition, the research also explored the decolorization of azo dye wastewater by immobilized functional bacteria DDMZ1 in the early stage. Based on these studies, this study improved the hydrolysis acidification process and analyzed its impact on the treatment of azo dye wastewater. In this study, the hydrolysis acidification process was enhanced to deeply treat azo dye wastewater by adding the highly efficient degradation functional bacterial agent DDMZ1 to the hydrolysis acidification aerobic reactor, adding co-metabolite fructose to the simulated azo dye wastewater, and immobilizing the functional bacterial group DDMZ1. By monitoring the decolorization ratio of wastewater and the COD (chemical oxygen demand) concentration of inlet and outlet water, the enhancement effect of each enhancement method on the treatment of azo dye wastewater by the hydrolysis acidification process is evaluated, and the optimal biological depth enhancement method is obtained to maximize the efficiency of the hydrolysis acidification process in the treatment of azo dye wastewater, which provides ideas for further improving the efficiency of the hydrolysis acidification process in practical engineering applications. In addition, this study also provides a strong theoretical basis for the hydrolysis acidification process in the treatment of other wastewater.

## 2. Results and Discussion

### 2.1. Strengthening Effect of Fructose Co-Matrix on Hydrolysis and the Acidification Process

#### 2.1.1. Decolorization and Degradation Effect of Bacteria without Fructose Co-Substrate

**Figure 1:**
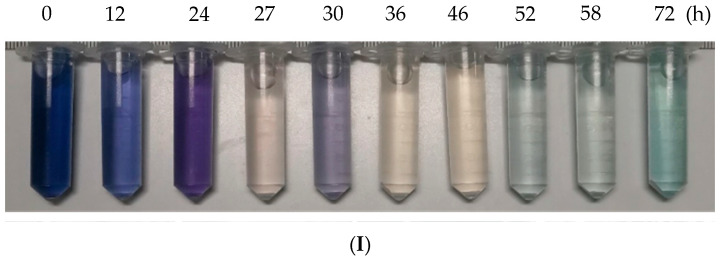

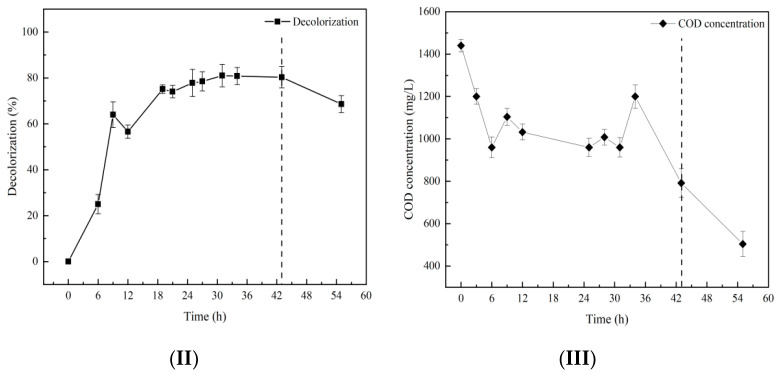
Treatment of simulated azo dye wastewater by the hydrolytic acidification aerobic reactor without fructose under intermittent operation. (**I**) decolorization effect diagram, (**II**) decolorization, (**III**) COD degradation. The vertical dotted line in the figure indicates the time point of starting aeration. The left side is the hydrolysis acidification stage and the right side is the aerobic stage.

After the simulated azo dye wastewater enters the reactor, the chromaticity and COD concentration of the wastewater is monitored, and photos are taken to record the actual situation of the wastewater treated by the reactor. The results are shown in Figure 1. It can be seen from Figure 1(I) that the color of the simulated azo wastewater in the early stage after entering the reactor gradually decreased, and it was close to clear and transparent at the 36th hour. At the 43rd hour of operation, the wastewater changed from the hydrolysis and acidification stage to the aerobic stage. After that, the color of the wastewater gradually changed from yellowish to light green. From the visual point of view, the color of the wastewater became darker, and a complex color appeared. It can be seen from Figure 1(II) that the decolorization ratio changed significantly in the first 12 h of the reactor operation, and the decolorization ratio reached 70% at the 10th hour, and then the decolorization ratio changed gradually. After the simulated azo dye wastewater entered the aerobic stage at 43 h, the decolorization ratio gradually decreased, which was consistent with the recolor phenomenon in the decolorization effect diagram.

As shown in Figure 1(III), the initial COD concentration of the wastewater was about (144 ± 100) mg/L, and the COD concentration of the wastewater gradually decreased with the action of the functional flora DDMZ1. After the hydrolysis acidification-aerobic reactor treatment, the COD concentration of the aerobic tank effluent was reduced to (504 ± 25) mg/L, and the COD degradation ratio reached 65%. From multiple sets of parallel experiments, the COD removal rate of simulated azo dye wastewater fluctuated from 32% to 65%. The hydrolytic acidification process can only degrade macromolecular organics into small molecule organics, but it is difficult to completely degrade them, so the COD removal rate is low. Ge Lei et al. [21] used hydrolysis acidification treatment as a pretreatment method to treat pharmaceutical wastewater and found that after more than 30 days of removal and more than one month of treatment, the removal rate of COD was only 40%.

#### 2.1.2. Strengthening Effect of Fructose Co-Matrix on the Hydrolysis-Acidification Process

**Figure 2:**
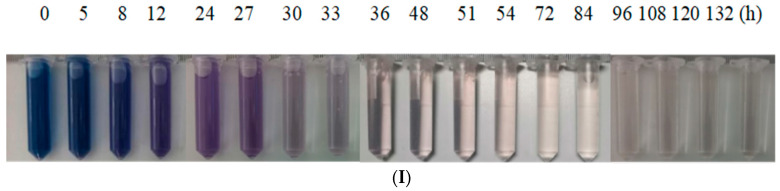

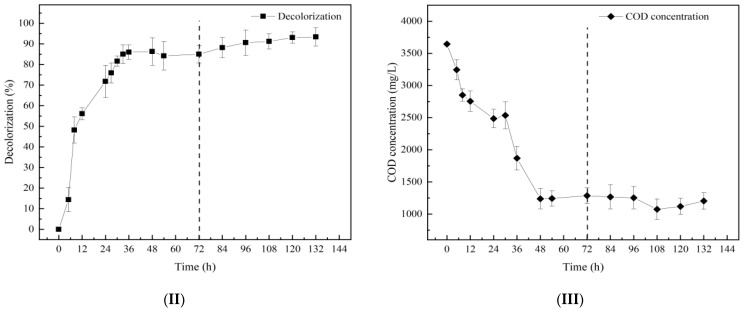
Fructose co-substrate enhanced hydrolysis-acidification aerobic reactor for the treatment of simulated azo dye wastewater under intermittent operation. (**I**) Decolorization effect diagram, (**II**) decolorization ratio, (**III**) COD degradation. The vertical dashed line in the figure indicates the time point when aeration was started. The left side is the hydrolytic acidification stage, and the right side is the aerobic stage.

It can be seen Figure 2(I) that with the operation of the reactor, the color of the wastewater in the experimental group with additional fructose co-substrate gradually became lighter, from dark blue to purple to nearly colorless. Figure 2(II), it can be seen that the decolorization ratio of wastewater increased significantly in the first 36 h of reactor operation, and the decolorization ratio reached 85% in the 36th hour, and then the change range became stable. After the wastewater enters the aerobic reaction stage, the decolorization ratio is further improved, reaching a maximum of 93%. As seen in Figure 2(III), the addition of fructose increased the initial COD concentration of the simulated azo dye wastewater to (3644 ± 37.28) mg/L. During the hydrolysis and acidification stage, the COD concentration showed a decreasing trend as a whole with a small fluctuation in the middle, which was similar to the change of the COD concentration in the experimental group without fructose. After entering the aerobic reaction stage, the COD concentration decreased rapidly in a short period and then gradually became stable. The COD concentration in the aerobic pool could reach the lowest (1075 ± 46.61) mg/L. The COD degradation ratio of wastewater can reach a maximum of 70%.

Compared to the experimental group without fructose addition, the group with additional fructose co-substrate showed a higher decolorization ratio. This indicates that the addition of the fructose co-substrate can strengthen the hydrolysis and acidification process, improve the biodegradability of wastewater, and increase the efficiency of simulated azo dye wastewater treatment. Moreover, in the experimental group with additional fructose co-substrate, no recolor phenomenon occurred when the wastewater entered the aerobic stage. This is speculated to be due to the presence of a fructose co-matrix, which further degrades the intermediate products of azo dyes, aromatic amines, and amides. Azo dye mineralization is more complete [22,23].

### 2.2. Strengthening Effect of Immobilization Technology on Hydrolysis and the Acidification Process

After determining the immobilization method, refer to the relevant literature for immobilization with sodium alginate, diatomaceous earth, and polyvinyl alcohol. The most suitable concentrations are: 1% sodium alginate and 7% polyvinyl alcohol, 4% sodium alginate and 1% diatomite [24]. The research group has tried these two methods in the early stage and evaluated the immobilization method from the bead forming state, mechanical strength, and stability of the immobilized functional flora DDMZ1 beads. Finally, 13% polyvinyl alcohol and 1% sodium alginate were selected.

**Figure 3:**
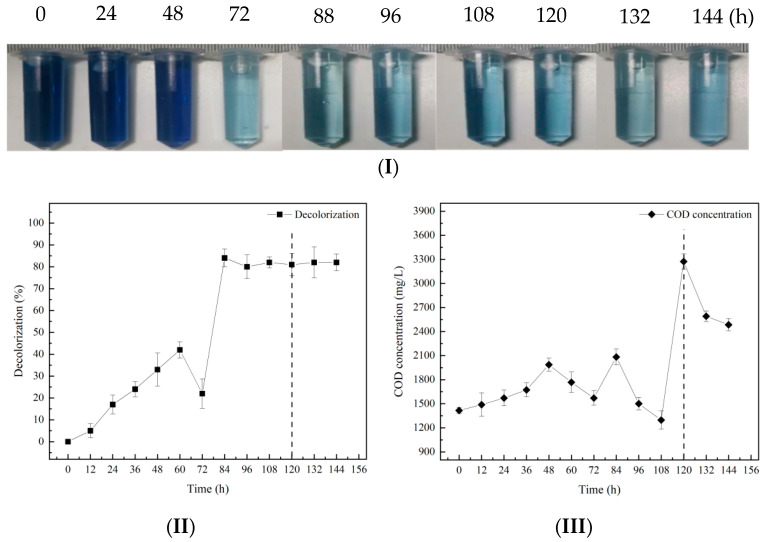
Effect of immobilized DDMZ1 on treatment of simulated azo dye wastewater by the hydrolytic acidification process under intermittent operation. (**I**) Decolorization effect diagram, (**II**) Decolorization of the first batch of wastewater, (**III**) COD degradation of the first batch of wastewater.

Combined with the decolorization effect diagram in Figure 3(I), it can be seen that the color of the wastewater did not change significantly during the first 48 h of the hydrolysis and acidification tank, which may be because the immobilized functional flora DDMZ1 was still in the adaptive growth phase, and the overall activity was not high. In the next 24 h, the color became lighter, from dark blue to light blue, indicating that the activity of the immobilized functional flora DDMZ1 gradually increased during this period and began to degrade the chromogenic substances in the wastewater. As the reaction progressed, the color of the wastewater remained relatively stable. At the 108th hour of the experiment, the wastewater entered the aerobic tank from the hydrolysis and acidification tank, the color of the wastewater became darker, and the phenomenon of re-coloring appeared. However, the decolorization ratio after re-coloring was still higher than before. From Figure 3(II), it can be observed that the decolorization ratio changed slowly in the early stage of hydrolysis and acidification, with a decolorization ratio of only about 34% at the 48th hour. Then, in the period of 72–88 h, the decolorization ratio increases rapidly and can reach up to 84%, which may be due to the adaptation of the immobilized functional flora DDMZ1 to the wastewater environment and its proliferation in the wastewater. This resulted in the degradation of a large amount of chromogenic substances. After the aerobic phase at 108 h, the decolorization ratio remained relatively constant at around 80%. In terms of COD concentration, it showed an overall upward trend during the entire process of using immobilized functional flora DDMZ1 to treat simulated azo dye wastewater. This may be related to the low microbial activity in the immobilized functional flora DDMZ1, resulting in less consumption of organic matter. In addition, the gradual precipitation of polyvinyl alcohol from the immobilized pellets during continuous wastewater treatment may also be a significant reason for the high COD concentration. Comparing the results with free functional flora DDMZ1, the use of immobilized functional flora DDMZ1 for the treatment of simulated azo dye wastewater improves the decolorization ratio and COD degradation ratio, indicating that the immobilized functional flora DDMZ1 technology can enhance the hydrolysis and acidification process of simulated azo dye wastewater. However, the issue of polyvinyl alcohol precipitation in the immobilized pellets needs to be urgently addressed.

### 2.3. Strengthening Effect of Co-Matrix Composite Immobilization Technology on Hydrolysis and the Acidification Process

**Figure 4:**
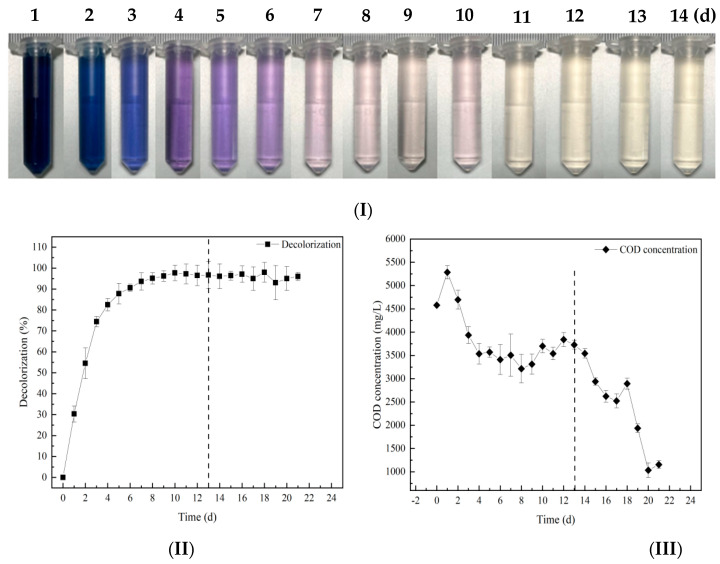
Effect of fructose co-matrix binding immobilized DDMZ1 on the treatment of simulated azo dye wastewater by the hydrolytic acidification process under batch operation under intermittent operation. (**I**) Decolorization effect diagram, (**II**) Decolorization, (**III**) COD degradation. The vertical dotted line in the figure indicates the time point of starting aeration. The left side is the hydrolysis acidification stage and the right side is the aerobic stage.

Previous studies have shown that a fructose co-substrate can improve the efficiency of hydrolysis and the acidification treatment of simulated azo dye wastewater. Based on this, the effect of the method of fructose co-matrix composite immobilization technology on the treatment of simulated azo dye wastewater in a hydrolysis acidification-aerobic reactor was studied. The results are shown in Figure 4. From the decolorization effect chart, the color of the wastewater gradually changed from dark blue to purple and light pink within 1–13 days and finally became close to colorless and transparent, remaining stable.

Combined with Figure 4(II), the decolorization ratio of simulated azo dye wastewater in the hydrolysis acidification tank first increased rapidly, and then gradually stabilized. On the 9th day, the decolorization ratio reached 97%. After the wastewater entered the aerobic tank from the hydrolysis and acidification tank on the 13th day, the decolorization ratio remained stable. For the COD concentration, when the pellets were first added, the COD increased, which may be caused by the precipitation of polyvinyl alcohol. Then, the COD concentration of the wastewater began to decrease from (4600.28 ± 114.97) mg/L to (3108.64 ± 86.18) mg /L, and the COD degradation ratio was 32%. It then entered a stable state. After the wastewater entered the aerobic stage, the COD concentration decreased significantly. After aeration for 7 days, the COD concentration decreased from (3661.28 ± 37.66) mg/L to (1011.36 ± 79.42) mg/L. The final COD degradation ratio could reach 78%.

Compared with the experiment of immobilization only, the decolorization effect of wastewater was better after adding fructose co-substrate. The decolorization ratio of the experimental group with additional fructose co-matrix was greatly improved, and the COD degradation was also greatly improved. This also indicated that the addition of fructose co-matrix had a certain positive effect on the immobilized functional flora DDMZ1.

### 2.4. Practical Application of Composite Bioaugmentation Technology

#### 2.4.1. Influence of Temperature on the Effect of Hydrolysis and the Acidification Process

**Figure 5:**
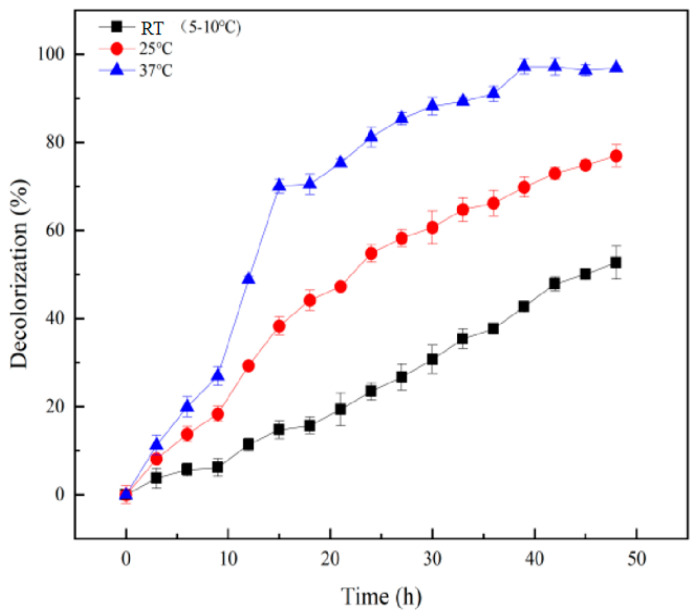
Decolorization of simulated azo dye wastewater treated by the hydrolysis acidification process at different temperatures.

In practical engineering applications, temperature can affect the efficiency of the entire wastewater treatment process and may even result in substandard effluent quality [25,26,27]. Figure 5 shows that the treatment effect of the hydrolysis acidification process on the azo dye wastewater is significantly different at different temperatures. At 37 °C, the decolorization ratio of wastewater was always higher than that of the experimental group at room temperature and 25 °C and reached a maximum of 96% at the 48th hour. Therefore, increasing the temperature can improve the efficiency of the hydrolysis-acidification process in treating the simulated azo dye wastewater. Current studies have shown that temperature will affect the activity of microorganisms, and each microorganism has an optimum growth temperature range [28]. The dominant genera in the functional flora DDMZ1 used in this study were *Lactococcus* and *Enterococcus*, and their optimum growth temperature was 37 °C, so the decolorization effect was best at 37 °C in this experiment.

Under the condition of winter temperatures of 5~10 °C in this experiment, the decolorization ratio of the simulated azo dyes within 48 h can still maintained at 50%, which indicates that the immobilized functional flora DDMZ1 complex fructose co-matrix has a hydrolysis effect even under low temperature conditions. The acidification process still had a strengthening effect, which further verifies the feasibility of this strengthening method in practical engineering applications.

#### 2.4.2. Influence of Salt Concentration on the Effect of Hydrolysis and the Acidification Process

**Figure 6:**
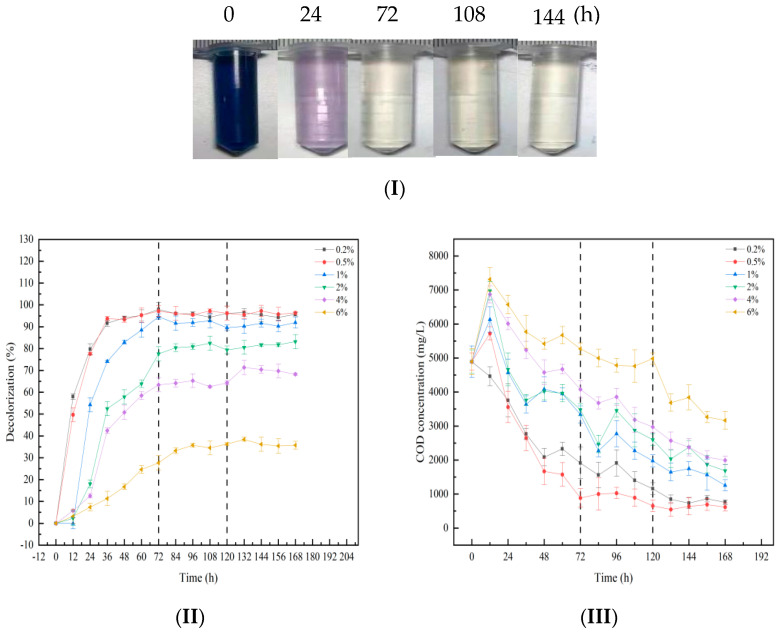
The treatment effect of the hydrolysis acidification process on azo dye wastewater with different salt concentrations. (**I**) Decolorization effect diagram, (**II**) Decolorization, (**III**) COD degradation. The five parallel centrifugal tubes in figure (**I**) are 0.2%, 0.5%, 1%, 2%, 4% and 6%, respectively from left to right. The black dotted line in figure (**II**) and figure (**III**) is the time point of starting aeration. The wastewater of 0.2% and 0.5% groups starts aeration at 72 h, and the wastewater of other groups starts aeration at 120 h.

It can be seen from Figure 6(I) that the wastewater with three concentrations of 0.2%, 0.5%, and 1% was close to a colorless and transparent state at 72 h, while the two kinds of wastewater with concentrations of 2% and 4% reached this state at 108 h. The color of the wastewater with a concentration of 6% was still darker at 144 h. This shows that the hydrolysis-acidification process has different treatment effects on simulated azo dye wastewater with different salt concentrations, and the treatment efficiency of the reactor gradually decreases with the increase in the salt concentration of simulated azo dye wastewater. It can also be seen from Figure 6(II) that decolorization ratio of the wastewater is lower with increasing salt concentration. The hydrolysis acidification-aerobic reactor can treat wastewater with salt concentrations of 0.2%, 0.5%, and 1% effectively, with a decolorization ratio of more than 90%. However, the decolorization ratio of wastewater with a salt concentration of 6% was only 38% at the highest. For COD degradation, the performance worsens with increasing salt concentration. The COD degradation ratios of the six batches of wastewater with salt concentrations of 0.2%, 0.5%, 1%, 2%, 4%, and 6% were 84%, 88%, 74%, 65%, 59%, and 35%, respectively. Among them, the wastewater with a salt concentration of 0.5% had the highest COD degradation ratio, and the decolorization ratio of the wastewater with salt concentrations of 0.2% and 0.5% did not differ significantly. It can be concluded that the salt concentration of the wastewater should not be too low, as it can improve the treatment efficiency of hydrolytic acidification.

In summary, the fructose co-matrix composite immobilization technology still has certain limitations in the hydrolysis and acidification treatment of high-salt printing and dyeing wastewater. To improve the degradation efficiency of high-salt wastewater, some microorganisms including halophilic bacteria can be isolated through domestication and then combined with technologies such as immobilization to treat the wastewater [29,30]. In addition, the salt concentration of azo dyes can also be reduced through pre-treatment, after which the wastewater can be treated. Membrane treatment is a commonly used desalination method of printing and dyeing wastewater is membrane treatment. Many membrane treatment technologies have been developed for wastewater desalination. For example, nano-TiO_2_ modified polyvinyl alcohol composite membrane has a high salt rejection rate in printing and dyeing wastewater [31].

#### 2.4.3. Effects of Different Dyestuffs Treated by Hydrolysis and the Acidification Process

**Figure 7:**
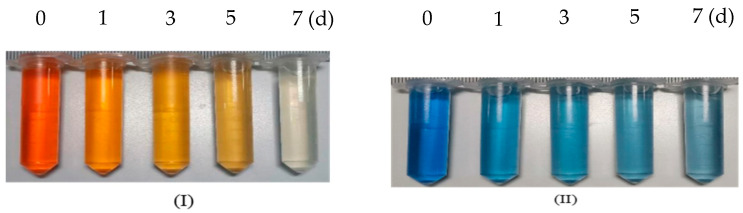

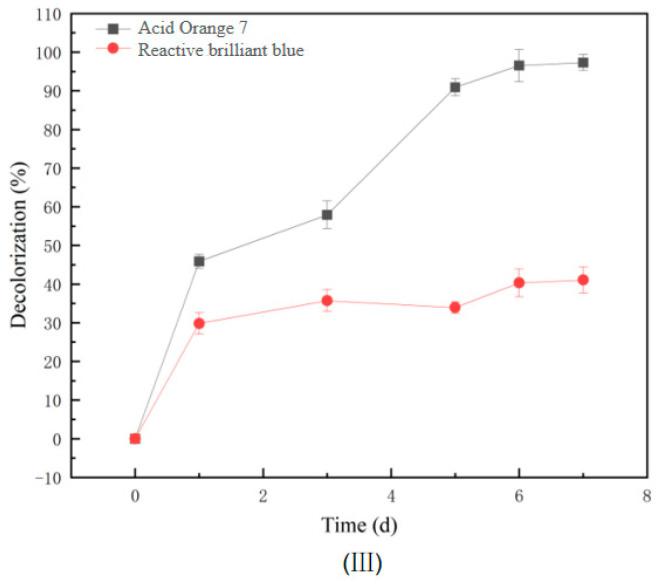
Treatment effect of fructose co matrix combined immobilization technology on acid orange 7 and reactive brilliant blue. (**I**) Decolorization effect of acid Orange 7, (**II**) Decolorization effect of reactive brilliant blue, (**III**) Change of decolorization ratio of two dyes.

Figure 7(I) shows the results of the hydrolysis and acidification treatment of acid orange 7 wastewater using the fructose co-matrix composite immobilization of functional flora DDMZ1 technology. It can be seen from the decolorization effect diagram that during the hydrolysis and acidification stage, the decolorization effect of the wastewater is remarkable. In the 0~7-day period, the wastewater gradually changes from orange to yellow and finally becomes close to colorless and transparent. When combined with the decolorization ratio change curve, it can be observed that the decolorization ratio reached 96% on the 6th day of the hydrolysis-acidification-aerobic reactor’s operation and remained stable on the 7th day. Comparing the treatment results of reactive black 5 dye wastewater by the same technology, the decolorization ratios of the two types of dye wastewater are similar, and the treatment of acid orange 7 wastewater can even reach a stable state faster. Therefore, the fructose co-matrix composite immobilization functional flora DDMZ1 technology has a good strengthening effect on the treatment of acid orange 7 wastewater by the hydrolysis and acidification process.

Figure 7(II) shows the results of the conditional hydrolysis and acidification of the fructose co-substrate complex immobilized functional bacteria DDMZ1-aerobic reactor for the treatment of reactive brilliant blue. It can be seen from the decolorization effect diagram that the reactive brilliant blue wastewater has a tendency to decolorize within 0~7 days, but the final state is still blue, and the chromaticity is much higher than that of acid orange 7. When combined with the change curve of the decolorization ratio, the overall decolorization ratio of wastewater is at a low level, and the decolorization ratio on the 7th day is only 44%, which is much lower than the decolorization ratio of active black 5 wastewater and acid orange 7 wastewater. From the dyes’ structures, both reactive black 5 and acid orange 7 belong to azo dye wastewater. The difference is that reactive black 5 has two azo bonds, while acid orange 7 has only one azo bond [32]. Reactive brilliant blue belongs to the anthraquinone dyes, which have a structure composed of two benzene rings and two carbonyl groups [33].

The results of this experiment show that the microorganisms in the functional flora DDMZ1 have selectivity for the degradation of dye wastewater, The degradation effect of azo dyes is relatively good, but the degradation effect of other structures is generally low. Therefore, the fructose co-matrix composite immobilized functional flora DDMZ1 technology used in this study is feasible for the treatment of azo dye wastewater by the hydrolysis and acidification process. On this basis, the immobilization technology can improve the effectiveness of treating other dye wastewater by strains with efficient degradation of other dyes, such as anthraquinone dyes.

## 3. Materials and Methods

### 3.1. Chemicals, Bacteria, and Cultivation Medium

RB5 was purchased from Sigma-Aldrich as a disazo acid dye. The artificial zeolite used in this study is chemically pure, while all other chemicals were of superior grade pure or analytical grade. The functional flora DDMZ1 used in this study was screened and isolated from the activated sludge in the secondary sedimentation tank of the domestic sewage treatment plant in Songjiang District during the early stage of the research group. It has an efficient degradation function for various azo dyes. The functional bacterial group DDMZ1 contains about 13 strains of different forms. SEM shows that most of the bacteria are rod-shaped, including short bacilli, long bacilli, *Fusobacterium*, etc. Its complex microbial community structure provides the possibility to degrade a variety of complex organic pollutants. The optimum pH and temperature for the growth of this bacterial group were 5.5 and 37 °C, respectively, which were suitable for static culture. The liquid basal medium for decolorizing experiments contained 0.50 g Na_2_SO_4_, 0.2 g NH_4_Cl, 2.66 g KH_2_PO_4_ and 3.00 g yeast extract per liter of distilled water. The medium was sterilized at high temperature and high pressure and then cooled for use.

### 3.2. Description of the Dye Wastewater Treatment System and Its Operations

**Figure 8:**
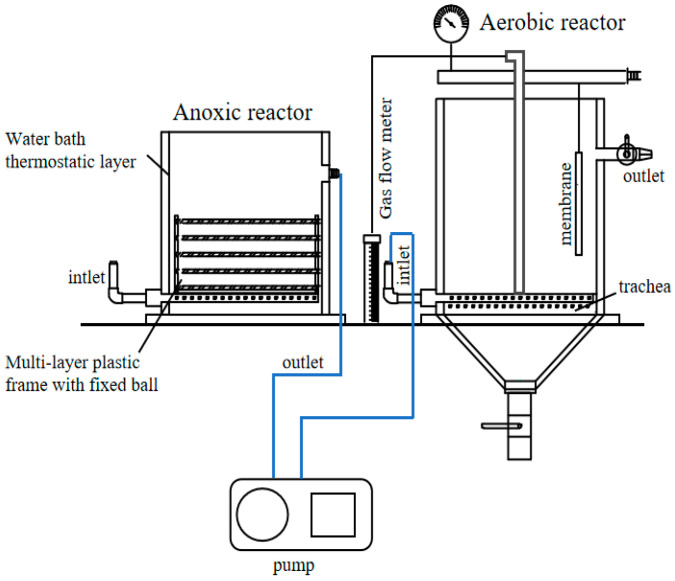
Schematic diagram of the biotreatment unit.

The reactor used in this experiment is a small-scale biotreatment device (produced by Shanghai Dayou Instrument Equipment Co., Ltd., Shanghai, China), which consists of a bacterial pool, aerobic pool, electromagnetic air compressor, lift pump, flow meter; its structure is shown in Figure 8. The reactor is divided into a hydrolysis acidification stage and an aerobic stage during operation, and the effective volumes of the hydrolysis acidification pool and the aerobic pool are both 3 L. At present, many studies on the treatment of printing and dyeing wastewater have confirmed that this is an effective biological treatment process. After this process, the COD degradation ratio can reach 80% to 90%, and the color of the wastewater can also be greatly reduced [34].

Intermittent operation: This operation is similar to the SBR sewage treatment process, including three stages of “water injection-reaction-drainage”. The wastewater is placed in the same reactor with an aeration device, which goes through the hydrolysis, acidification stage, and aeration stage successively, and there is no new wastewater in the treatment process. The operation mode is simple in operation and flexible in control. When the treatment effect is not good, it can be adjusted at any time by adding functional bacteria agent, extending the hydraulic residence time, and other means. In this study, the water inlet mode of intermittent operation was set up to initially explore the influence of various factors on the hydrolysis and acidification treatment of simulated azo dye wastewater, so as to facilitate better continuous operation in the future.

### 3.3. Intensification of the Hydrolysis-Acidification Process by a Fructose Co-Matrix

Under intermittent operating conditions, the simulated azo dye wastewater in the treatment group without fructose co-substrate was composed of 2.66 g/L KH_2_PO_4_, 0.2 g/L NH_4_Cl, 0.5 g/L Na_2_SO_4_, 0.1 g/L reactive black 5 dye, and 3 g/L yeast extract composition. The fructose co-substrate treatment group was supplemented with 3 g/L fructose on this basis. Three liters of simulated azo dye wastewater was added the hydrolysis and acidification tank, and then add 300 mL of DDMZ1, a high-efficiency degradable bacterial group that had been cultivated to the logarithmic growth phase in advance (the dosage ratio of simulated azo dye wastewater to bacterial agent was 1 L: 100 mL) was added. The chromaticity and COD of the wastewater were regularly monitored. After the chromaticity of the simulated azo dye was reduced and maintained for a while, the pump connecting the hydrolysis acidification tank and the aerobic tank was turned on, so that all the wastewater entered the aerobic tank. The air compressor was turned on for aeration. During the aeration stage, the chromaticity and COD concentration of the wastewater were irregularly monitored, and three parallel samples were selected for determination under the same conditions. The experimental group without fructose and the experimental group with additional fructose co-substrate were run 3–4 times respectively. Finally, the data from the two groups of experiments were plotted, analyzed, and compared. By comparing the difference between the two groups of experimental data, it could be concluded that fructose, as a co-substrate, enhanced the effect of the hydrolysis-acidification process on the treatment of simulated azo dye wastewater.

### 3.4. Strengthening Immobilization Technology 

#### 3.4.1. Enhancement of Hydrolysis and the Acidification Process by Functional Flora Immobilization

After exploring various immobilization methods, it was decided to use 13% polyvinyl alcohol and 1% sodium alginate to immobilize the functional flora DDMZ1. Other reagents such as zeolite, PBS (phosphate-buffered solution), cross-linking agent, and embedding solution were also used.

The specific steps for immobilization were as follows [35]: Take 1 mL of functional flora DDMZ1 in the logarithmic growth phase, centrifuge it at 8000 rpm for 10 min, wash it twice with PBS buffer, adjust the OD600 value to about 0.7, and add an appropriate amount of zeolite suspension to absorb bacteria. Pour the bacterial solution into the embedding solution at about 37 °C and stir it evenly in a water bath at 37 °C. Prepare 60 mL of cross-linking agent, several beakers, and use a 1 mL syringe to suck the embedding solution added to the bacterial cells, about 10 cm away from the cross-linking agent, drop by drop at a uniform speed. Seal and cross-link at 4 °C for 12–24 h. After cross-linking, rinse the pellets 2–3 times with sterile saline and store them in a 4 °C refrigerator for later use.

**Figure 9:**
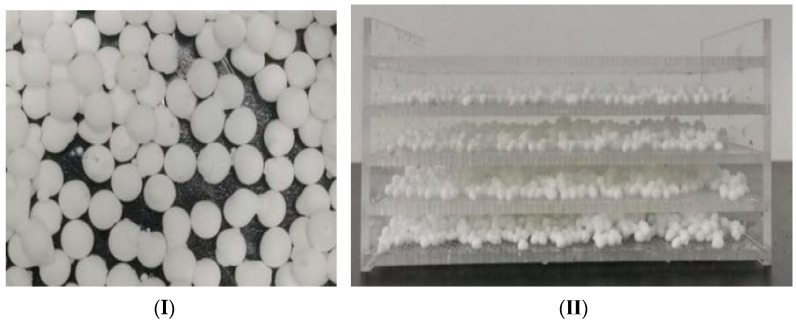
(**I**) Immobilized balls of functional bacteria DDMZ1, (**II**) immobilized balls of functional bacteria DDMZ1on multi-layer plastic frame.

After obtaining the immobilized functional flora DDMZ1, it was necessary to increase the contact area between the immobilized functional flora DDMZ1 and the wastewater as much as possible. Therefore, it was laid on a multi-layer plastic frame, as shown in Figure 9, and then put into the hydrolysis acidification tank along with the plastic plate frame. The experimental system was divided into two groups for control. One group was placed in the hydrolysis acidification tank with immobilized functional bacteria DDMZ1, and the other group was added with free functional bacteria agent DDMZ1. By monitoring the chromaticity and COD concentration of the effluent of the two groups of the experimental hydrolysis acidification tanks and aerobic tanks, the enhancement effect of the immobilized functional flora DDMZ1 technology on the treatment of simulated azo dye wastewater by the hydrolysis acidification process was evaluated.

#### 3.4.2. Study on the Hydrolysis and Acidification Effect of Functional Flora DDMZ1 Enhanced by Co-Matrix Composite Immobilization Technology

After exploration of the preparation method of immobilized functional flora DDMZ1, the effect of two methods of fructose co-substrate composite immobilization technology on the treatment of simulated azo dye wastewater by hydrolysis-acidification-aerobic reactor was studied. Similarly, the experimental systems were divided into two groups as control groups. One group added fructose co-substrate in the hydrolysis-acidification pool with immobilized functional bacteria DDMZ1, and the other group did not add co-substrate. During the experiment, the chromaticity and COD concentration in the hydrolysis-acidification tank and the aerobic tank were regularly detected, and the decolorization ratio and COD degradation ratio of the wastewater were calculated. The hydrolysis-acidification process was used to deeply strengthen the effect of simulated azo dye wastewater.

### 3.5. Exploration of the Practical Application of Compound Bioaugmentation Technology

#### 3.5.1. The Effect of Temperature on the Effect of Enhanced Hydrolysis and Acidification of Immobilized Functional Flora DDMZ1

During the entire experimental period of this study, the decolorization ratio of wastewater changed with the variation in ambient temperature, indicating that temperature has a significant influence on the efficiency of the hydrolysis-acidification process. Since the ambient temperature will change with the climate in the actual wastewater treatment process, this study explored the effect of different temperatures on the treatment of simulated azo dyes by the hydrolysis-acidification process. In this experiment, the intermittent operation mode was selected, and other conditions were kept constant. Under the condition of winter room temperature, the temperature of the water bath was adjusted by turning on the water bath circulating pump, and three different temperature gradients were set, namely low temperature 5~10 °C (room temperature), normal temperature 25 °C, and 37 °C sequentially. Similarly, the effect of temperature on the hydrolysis-acidification-aerobic reactor treatment of simulated azo dye wastewater was evaluated by monitoring the decolorization ratio of simulated printing and dyeing wastewater during the reaction.

#### 3.5.2. Effect of Salt Concentration on the Effect of Enhanced Hydrolysis and Acidification of Immobilized Functional Bacteria DDMZ1

In the process of dyeing with dyes, a large number of inorganic salts (30~90 g/L), such as sodium sulfate and sodium chloride, can be used to improve the affinity of dyes for fibers, but they do not participate in any chemical reactions [36,37]. However, up to 60% of the inorganic salts will enter the wastewater after the dyeing process, which often leads to high salt concentrations in practical printing and dyeing wastewater [38]. Therefore, salt concentration has become an important factor in exploring the practical application of hydrolysis-acidification process enhancement technology. In this study, wastewater with different salt concentrations (mass fraction) was prepared by adding NaCl to the simulated azo dye wastewater, and the batch operation mode was adopted, combined with fructose co-substrate composite immobilization technology to treat the simulated azo dye wastewater with different salt concentrations. By monitoring the decolorization ratio and COD concentration of the wastewater, the effect of bioaugmentation technology on the treatment of wastewater with different salt concentrations by the hydrolysis-acidification process was evaluated.

#### 3.5.3. Research on the Decolorization Effect of Fructose Co-Matrix Composite Immobilization Technology on Other Dye Wastewater

Two other dyes, acid orange 7 and reactive brilliant blue, were selected for this study. Acid orange 7 is a monoazo dye, also known as 2-naphthol orange, while reactive brilliant blue is an anthraquinone dye. The same approach was used to treat both wastewaters. By comparing the treatment effect of the wastewater, the deep strengthening effect of fructose co-matrix composite immobilization technology on the treatment of different dye wastewater by hydrolysis acidification process was explored. The simulated dye wastewater consists of 2.66 g/L KH_2_PO_4_, 0.2 g/L NH_4_Cl, 0.5 g/L Na_2_SO_4_, 0.1 g/L acid orange 7 (reactive brilliant blue) dye, 3 g/L yeast extract, and 3 g/L fructose. The decolorization ratio is one of the indicators that can best reflect the treatment effect of dye wastewater. In this experiment, the treatment effect of the hydrolysis-acidification-aerobic reactor on wastewater was evaluated by regularly monitoring the decolorization ratio of wastewater.

### 3.6. Experimental Evaluation Index

When evaluating the efficiency of the hydrolysis-acidification process to treat azo dye wastewater, it is particularly important to select appropriate indicators for evaluation. Azo dye wastewater has high chroma and high concentrations of organic pollutants. Therefore, in this study, the decolorization ratio of wastewater and the degradation ratio of COD concentration were selected to evaluate the effect of the hydrolysis-acidification process on simulated azo dye wastewater.

#### 3.6.1. Decolorization Studies

Take 2 mL of water sample and centrifuge at 8000 rpm for 10 min. After centrifugation, take the supernatant to measure the absorbance at 597 nm. The decolorization ratio was calculated using Equation (1) as follows:

(1) $$\mathrm{Decolorization}\left(\%\right)=\frac{\left(B-A\right)}{B}\times 100\%$$

where B is the initial absorbance and A is the absorbance of the degraded medium.

#### 3.6.2. COD Assay

COD is determined by microwave digestion method using a microwave digestion instrument.

## 4. Conclusions

The study found that adding fructose co-substrate as a co-metabolite to the simulated azo dye wastewater could improve the decolorization ratio and COD degradation ratio of wastewater. After 42 h of hydrolysis and acidification treatment, the decolorization rate and COD degradation ratio of the experimental group with additional fructose co-substrate could reach 93% and 70%, respectively, while the decolorization rate and COD degradation ratio of the wastewater without fructose addition group were only 78% and 65%, respectively. Additionally, the immobilized functional bacteria DDMZ1 technology can also improve the hydrolysis and acidification process. Moreover, in the process of using the hydrolysis acidification-aerobic reactor to treat the simulated azo dye wastewater, the fructose co-matrix composite immobilization functional flora DDMZ1 technology has a better performance than the fructose co-matrix enhancement and immobilization functional flora DDMZ1 technology alone. The combination of the two enhanced technologies enables wastewater to decolorize on the first day of reactor operation and achieves the best effect on the ninth day, with a maximum decolorization rate of 97%. Furthermore, through the exploration of practical applications, it was found that the environmental temperature and the salt concentration of the simulated azo dye wastewater will affect the efficiency of the hydrolysis acidification process to treat the simulated azo dye wastewater. Additionally, it was also found that the decolorization effect of azo dye wastewater by using fructose co-matrix composite immobilization technology is better, but the decolorization effect of anthraquinone dye wastewater is poor.

## Data Availability

The data presented in this study are available on reasonable request from the corresponding author.

## References

[1] Sequin-Frey (1981). The chemistry of plant and animal dyes. J. Chem. Educ..

[2] Zuorro A., Lavecchia R., Medici F., Piga L. (2013). Spent tea leaves as a potential low-cost adsorbent for the removal of azo dyes from wastewater. Chem. Eng..

[3] Forgacs E., Cserhati T., Oros G. (2004). Removal of synthetic dyes from wastewaters: A review. Environ. Int..

[4] Gupta V.K., Kumar R., Nayak A., Saleh T.A., Barakat M.A. (2013). Adsorptive removal of dyes from aqueous solution onto carbon nanotubes: A review. Adv. Colloid Interface Sci..

[5] Elizalde-González M.P., Arroyo-Abad U., García-Díaz E., Brillas E., Sirés I., Dávila-Jiménez M.M. (2012). Formation of sulfonyl aromatic alcohols by electrolysis of a bisazo reactive dye. Molecules.

[6] Gičević A., Hindija L., Karačić A. (2019). Toxicity of azo dyes in pharmaceutical industry. Proceedings of the International Conference on Medical and Biological Engineering.

[7] Zeng Q., Wang Y., Zan F., Khanal S.K., Hao T. (2021). Biogenic Sulfide for Azo Dye Decolorization from Textile Dyeing Wastewater. Chemosphere.

[8] Petrucci E., Di Palma L., Lavecchia R., Zuorro A. (2015). Treatment of diazo dye Reactive Green 19 by anodic oxidation on a boron-doped diamond electrode. J. Ind. Eng. Chem..

[9] Khaled J.M., Alyahya S.A., Govindan R., Chelliah C.K., Maruthupandy M., Alharbi N.S., Kadaikunnan S., Issac R., Murugan S., Li W.-J. (2022). Laccase producing bacteria influenced the high decolorization of textile azo dyes with advanced study. Environ. Res..

[10] Xu H., Yang B., Liu Y., Li F., Shen C., Ma C., Tian Q., Song X., Sand W. (2018). Recent advances in anaerobic biological processes for textile printing and dyeing wastewater treatment: A mini-review. World J. Microbiol. Biotechnol..

[11] Vojnović B., Cetina M., Franjković P., Sutlović A. (2022). Influence of initial pH value on the adsorption of reactive black 5 dye on powdered activated carbon: Kinetics, mechanisms, and thermodynamics. Molecules.

[12] Wang Y., Zhu K., Zheng Y., Wang H., Dong G., He N., Li Q. (2011). The effect of recycling flux on the performance and microbial community composition of a biofilm hydrolytic-aerobic recycling process treating anthraquinone reactive dyes. Molecules.

[13] Wang H., Li Q., He N., Wang Y., Sun D., Shao W., Yang K., Lu Y. (2009). Removal of anthraquinone reactive dye from wastewater by batch hydrolytic–aerobic recycling process. Sep. Purif. Technol..

[14] Na L., Xuehui X., Fang Y., Lewei S., Chengzhi Y., Jianshe L. (2016). Effect of bacterial agent strengthening on hydrolysis acidification treatment of simulated printing and dyeing wastewater. J. Environ. Eng..

[15] Khan Z., Jain K., Soni A., Madamwar D. (2014). Microaerophilic degradation of sulphonated azo dye—Reactive Red 195 by bacterial consortium AR1 through co-metabolism. Int. Biodeterior. Biodegrad..

[16] Li L., Zhang M., Jiang W., Yang P. (2022). Study on the efficacy of sodium alginate gel particles immobilized microorganism SBBR for wastewater treatment. J. Environ. Chem. Eng..

[17] Balapure K.H., Jain K., Chattaraj S., Bhatt N.S., Madamwar D. (2014). Co-metabolic degradation of diazo dye—Reactive blue 160 by enriched mixed cultures BDN. J. Hazard. Mater..

[18] Zhang Q., Xie X., Xu D., Hong R., Wu J., Zeng X., Liu N., Liu J. (2021). Accelerated azo dye biodegradation and detoxification by Pseudomonas aeruginosa DDMZ1-2 via fructose co-metabolism. Environ. Technol. Innov..

[19] Zhang X., You S., Ma L., Chen C., Li C. (2015). The application of immobilized microorganism technology in wastewater treatment. Proceedings of the 2015 2nd International Conference on Machinery, Materials Engineering, Chemical Engineering and Biotechnology.

[20] D’Souza S. (2002). Trends in immobilized enzyme and cell technology. Indian J. Biotechnol..

[21] Lei G., Ren H., Ding L., Wang F., Zhang X. (2010). A full-scale biological treatment system application in the treated wastewater of pharmaceutical industrial park. Bioresour. Technol..

[22] Liu N. (2016). Study on the Change of Microbial Community Structure and Its Optimal Regulation in the Process of Hydrolysis Acidification Treatment of Printing and Dyeing Wastewater. Ph.D. Thesis.

[23] Liu N., Lin Z., Xie X., Sun P., Wang L., Su H. (2020). Effect of oxygen on decolorization and degradation of azo dyes by mixed bacteria. Bull. Microbiol..

[24] Wang L., Luo Q.-F. (2006). Biodegradation of dibutyl phthalate by diatomite adsorptive immobilized microorganism. Wei Sheng Yan Jiu J. Hyg. Res..

[25] Junnan S., Kai C., Haiyan L. (2021). Winter Operation and Management Practice of Comprehensive Sewage Treatment Plant in Chemical Industry Park. Water Supply Drain..

[26] Meng C., Ruifen M. (2016). Analysis of biological nitrogen and phosphorus removal effect of WWTP in winter operation. China Water Supply Drain..

[27] Fidaleo M., Lavecchia R., Petrucci E., Zuorro A. (2016). Application of a novel definitive screening design to decolorization of an azo dye on boron-doped diamond electrodes. Int. J. Environ. Sci. Technol..

[28] Imran M., Crowley D.E., Khalid A., Hussain S., Mumtaz M.W., Arshad M. (2015). Microbial biotechnology for decolorization of textile wastewaters. Rev. Environ. Sci. Biol. Technol..

[29] He H., Chen Y., Li X., Cheng Y., Yang C., Zeng G. (2017). Influence of salinity on microorganisms in activated sludge processes: A review. Int. Biodeterior. Biodegrad..

[30] Guo J., Zhou J., Wang D., Tian C., Wang P., Uddin M.S. (2008). A novel moderately halophilic bacterium for decolorizing azo dye under high salt condition. Biodegradation.

[31] Li X., Chen Y., Hu X., Zhang Y., Hu L. (2014). Desalination of dye solution utilizing PVA/PVDF hollow fiber composite membrane modified with TiO_2_ nanoparticles. J. Membr. Sci..

[32] Méndez-Paz D., Omil F., Lema J.M. (2005). Anaerobic treatment of azo dye Acid Orange 7 under batch conditions. Enzym. Microb. Technol..

[33] Liu Y., Hua L., Li S. (2010). Photocatalytic degradation of reactive brilliant blue KN-R by TiO_2_/UV process. Desalination.

[34] Yang C., Tong Z., Gang C., Jiang T. (1999). Study on the treatment process of printing and dyeing wastewater. J. Northwest Text. Inst. Technol..

[35] Xiaolei W., Jianguang L., Xiang H., Yuxin Y. (1993). Studies on Sodium Alginate and Polyvinyl Alcohol as Immobilized Microbial Embedding Agents. Environ. Sci..

[36] Nallathambi A., Rengaswami G.D.V. (2016). Salt-free reactive dyeing of cotton hosiery fabrics by exhaust application of cationic agent. Carbohydr. Polym..

[37] Buscio V., López-Grimau V., Álvarez M.D., Gutiérrez-Bouzán C. (2019). Reducing the environmental impact of textile industry by reusing residual salts and water: ECUVal system. Chem. Eng. J..

[38] Hu E., Shang S., Tao X.M., Jiang S., Chiu K.L. (2016). Regeneration and reuse of highly polluting textile dyeing effluents through catalytic ozonation with carbon aerogel catalysts. J. Clean. Prod..

